# Pacific‐Led Responses to COVID‐19: Lessons for Future Pandemic Preparedness

**DOI:** 10.1002/snz2.70049

**Published:** 2026-04-26

**Authors:** Amio Matenga‐Ikihele, Fale Asafo, Ruby Tuesday, Natalie Netzler, Chris Puliuvea, Teuila Percival

**Affiliations:** ^1^ Moana Connect Auckland New Zealand; ^2^ Pacific Health Section Faculty of Medical and Health Sciences University of Auckland Auckland New Zealand; ^3^ Faculty of Medical and Health Sciences The University of Auckland Auckland New Zealand; ^4^ Biomedicine and Medical Diagnostics School of Science Auckland University of Technology Auckland New Zealand

**Keywords:** COVID‐19, Pacific peoples, pandemic, preparedness

## Abstract

The COVID‐19 pandemic exposed deep inequities in health systems globally and in Aotearoa New Zealand, with Pacific communities experiencing a disproportionate burden of illness, economic hardship, and social disruption. Despite these challenges, Pacific communities demonstrated resilience, culturally grounded leadership, and the ability to meet community needs through collective action. This qualitative review of peer‐reviewed literature, government reports, and community‐led research identified five interconnected themes: (1) community partnerships; (2) Pacific‐centred approaches; (3) clear and trusted communication; (4) digital inclusion and literacy skills; and (5) economic support and sustainability. From these themes, key enablers were identified, which included community leadership, trusted communication strategies, and agile local systems, alongside barriers such as underinvestment, digital exclusion, reliance on unpaid labour, and limited inclusion of Pacific leadership in early planning. The findings highlight that Pacific‐led systems are not supplementary but an essential public health infrastructure. Embedding these approaches within national emergency planning, through sustainable funding, formal governance roles, and strengthened digital inclusion, offers a pathway to a more equitable, trusted, and resilient pandemic response.

## 1 Introduction

The COVID‐19 pandemic had a profound impact on Pacific peoples living in Aotearoa New Zealand, disrupting their collective way of life and intensifying pre‐existing inequities (Te Hiringa Mahara – Mental Health and Wellbeing Commission 2023). Pacific peoples collectively refer to the diverse communities originating from the many nations and territories of Oceania, spanning Polynesia, Melanesia, and Micronesia, each with their own distinct languages, cultures, and histories (Ministry for Pacific Peoples 2022). As a community with a long history of migration and settlement in Aotearoa New Zealand, Pacific peoples today number 442,632, comprising 8.9% of the total population (Ministry of Health 2025). The seven largest Pacific groups in Aotearoa are Samoan, Tongan, Cook Island, Niuean, Fijian, Tokelauan, and Tuvaluan, with the majority residing in the Auckland region (Ministry for Pacific Peoples 2022). Despite this established presence, Pacific peoples continue to experience significant health and social inequities, disparities that the pandemic both exposed and deepened (Ministry of Health 2025).

Between 2020 and 2023, Pacific peoples recorded the highest COVID‐19 hospitalisation rate in the country, with 17 hospitalisations per 1000 cases compared to 11 per 1000 for the total population (Steyn et al. 2020; Reddy et al. 2023; Te Poutoko Ora a Kiwa 2024). These outcomes were compounded by socioeconomic factors such as overcrowded housing, limited access to culturally appropriate healthcare, and the prevalence of multigenerational households, which heightened transmission risk. Systemic barriers, such as inaccessible language, cost, and mistrust of health services, further increased vulnerability during the pandemic (Te Hiringa Mahara – Mental Health and Wellbeing Commission 2023; Steyn et al. 2020). Service gaps widened markedly for Pacific communities, particularly in planned care, immunisation coverage, cancer screening, and children's oral health assessment and treatment (Te Whatu Ora 2022).

Early in the pandemic, the World Health Organization stressed that effective public health interventions depend on the combined efforts of government action and active community participation (WHO 2020). In Aotearoa, Pacific communities, churches, and health providers embodied this principle, drawing on cultural values such as voluntary service to meet community needs. Many mobilised quickly to deliver culturally tailored responses that addressed both immediate and longer‐term needs that impact wellbeing. These efforts included food distribution, mobile vaccination clinics, culturally safe care, digital inclusion initiatives, and ensuring health information was shared in ways that were meaningful and accessible. Their effectiveness lay in being firmly grounded in Pacific values of collectivism, service, and spirituality (Ministry of Health 2021).

There remains much to learn from the pandemic experiences of Pacific communities in Aotearoa, New Zealand, with a more comprehensive understanding needed to inform future responses that are equitable and culturally grounded. The aim of this review was to examine Pacific‐led responses to the COVID‐19 pandemic in Aotearoa New Zealand. It sought to identify the key enablers and barriers influencing the effectiveness of these responses, highlight gaps in current preparedness efforts, and provide recommendations to strengthen future pandemic responses for Pacific communities.

## 2 Methods

This study used a qualitative synthesis approach to identify and evaluate literature related to Pacific‐led responses and the COVID‐19 pandemic in Aotearoa New Zealand. This review included academic and grey literature published between 2020 and 2024, with a primary focus on COVID‐19 and Pacific communities in Aotearoa New Zealand.

**FIGURE 1:**
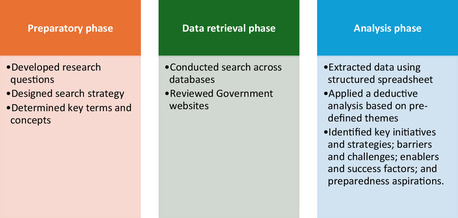
Search methods employed in this study to examine the COVID‐19 pandemic response and experiences for Pacific communities in Aotearoa, New Zealand published between 2020 and 2024.

Using a three‐phase process (Figure 1), the review incorporated a range of sources, including peer‐reviewed journal articles, government and health sector reports, and community‐led evaluations focused on Pacific populations.

### 2.1 Preparatory Phase

The team designed a targeted search strategy to identify literature and documentation examining Pacific responses to the COVID‐19 pandemic. To be included, sources were required to meet the following criteria:

1. A focus on Pacific communities or Pacific‐led organisations.
2. Detailed descriptions of pandemic or infectious disease responses, with particular emphasis on COVID‐19.
3. Publications dated between 2020–2024, with the COVID‐19 pandemic as a primary focus.

To be included, sources needed to document responses specific to Pacific populations during the COVID‐19 pandemic. Sources that did not discuss Pacific contexts or lacked relevance to pandemic preparedness or responses were excluded.

### 2.2 Data Retrieval Phase

Searches were undertaken across a range of academic databases (e.g., Scopus, PubMed), official New Zealand government repositories, and websites of Pacific health providers. Supplementary sources were identified through targeted searches of community organisation platforms and reviewing reference lists from relevant publications. The search was guided by targeted keywords and phrases including but not limited to: “Pacific pandemic preparedness,” “Pacific health response,” “COVID‐19 Pacific response,” “Pacific infectious disease management,” “COVID‐19 capability,” “Pacific infectious disease response,” and “Pacific and COVID‐19.”

### 2.3 Analysis Phase

The review analysed 20 documents, including peer‐reviewed journal articles, government and health sector reports, and community‐led research. A deductive coding approach was undertaken by two researchers to systematically analyse the documents. Each source was reviewed and coded within a structured matrix developed in Microsoft Excel. Documents and articles were listed along the *Y*‐axis, while predefined analytical categories, aligned with the research questions, were arranged along the *X*‐axis. These categories focused on four key areas: (1) key themes underpinning Pacific‐led pandemic responses; (2) challenges encountered during the COVID‐19 response; (3) enablers that supported the response; and (4) aspirations to strengthen future pandemic planning and responses.

Once coding was complete, the two researchers reviewed the coded data across all documents to identify ideas and concepts that appeared repeatedly. Concepts that were similar in nature were grouped together, and through discussion, the researchers agreed on five overarching themes that best captured the findings across the literature.

## 3 Results

A thematic synthesis of the evidence identified five interrelated themes in Pacific‐led responses to COVID‐19: (1) community partnerships; (2) Pacific‐centred approaches; (3) clear and trusted communication; (4) digital inclusion and literacy skills; and (5) economic support and sustainability. Each theme explores both the enablers that supported effective response efforts and the barriers that hindered them, offering critical insights to inform future pandemic preparedness and response strategies. A summary of these key themes is provided in Table 1.

**TABLE 1:** Summary of key themes from articles and reports on Pacific responses to the COVID‐19 pandemic in Aotearoa, New Zealand.

Theme	Enablers and Success Factors	Barriers and Challenges
**Community resilience**	Established relationships and trust; Strong cultural values and collective action; Church and community leadership; Pacific leadership embedded in planning; Pacific emergency response teams; Churches as hubs for essential aid and spiritual care; Youth digital support; Swift mobilisation via community networks.	Reliance on volunteers and short‐term funding; Under‐resourced organisations; Digital exclusion; Limited health service access; Remote learning challenges; Unequal impacts of specific COVID‐19 variants (e.g., the Delta variant) Restrictions on in‐person cultural and funeral practices.
**Pacific centred approaches**	Trusted Pacific providers; Pacific‐led initiatives grounded in culture; Trusted leaders delivering spiritual, emotional, and practical care; Culturally grounded outreach using Pacific languages and talanoa; Integration of holistic models of care.	Institutional racism; Absence of culturally responsive planning; Inadequate resourcing of Pacific initiatives; Fragmentation of mainstream responses.
**Clear and trusted communications**	PreparePacific.nz platform; Faith‐based communication channels; Messages reframed through cultural values; Talanoa sessions to address misinformation; Pacific languages and youth‐led digital engagement.	Lack of clear and culturally relevant information; Limited translated materials; Digital access issues; Widespread misinformation, especially online.
**Digital inclusion and literacy skills**	Youth‐elder pairing for tech support; Churches facilitating online services; Digital inclusion framed as a structural health determinant; Pacific‐led digital literacy programmes like DIGIFALE.	Limited access to devices and internet; Low digital literacy; Reliance on in‐person services; Intergenerational gaps in digital engagement.
**Economic support and sustainability**	Churches and community groups distributing essentials; Sustainable practices like community gardening; Parenting and economic resilience programmes; Government subsidies; strategic preparedness in churches; Longer‐term structural investment.	Job losses, housing insecurity, high living costs; Short‐term and fragmented government funding; Over‐reliance on volunteer staff and underfunded providers.

### 3.1 Community Partnerships

Community‐led initiatives were most effective when genuine partnerships existed between Pacific communities, government agencies, and other stakeholders. These relationships, particularly those built on long‐standing trust and accountability, enabled timely and targeted delivery of welfare support, public health messaging, and essential services to vulnerable families (Ioane et al. 2021; Ministry for Pacific Peoples 2021; Moana Connect 2024; Te Hiringa Mahara – Mental Health and Wellbeing Commission 2023). Pacific communities' ability to mobilise quickly, particularly in the early phases of the pandemic, was underpinned by their community networks and relational leadership. In several reports, Pacific communities leveraged their own resources to provide food, hygiene supplies, and emotional support, often bridging the gap between government agencies and community need (Ministry for Pacific Peoples 2021; Te Hiringa Mahara – Mental Health and Wellbeing Commission 2023). Several organisations, with support from Health New Zealand/Te Whatu Ora, used a “Manaaki Care Approach” to deliver aid, combining cultural leadership with practical assistance. This included the development of dedicated Pacific emergency response teams and community‐led initiatives that were agile and responsive to local needs (Moana Connect 2024).

Many churches developed their own COVID‐19 response plans and became hubs for distributing essential services such as food parcels, hygiene packs, especially to families who were isolating, had lost work, or were too scared to leave home (Ministry for Pacific Peoples 2021; Pasifika Proud 2021; Te Hiringa Mahara – Mental Health and Wellbeing Commission 2023). Even when in‐person gatherings were restricted, many churches‐maintained connexion through online church services, with ministers and church leaders providing spiritual and emotional support to their congregations (Ministry for Pacific Peoples 2021;Te Hiringa Mahara – Mental Health and Wellbeing Commission 2023). Youth played an instrumental role in facilitating digital engagement, ensuring older family members remained informed and socially connected (Ministry for Pacific Peoples 2021;Matenga‐Ikihele et al. 2023).

Although healthcare services continued to operate during the pandemic, Pacific communities identified five key barriers to accessing health, social, and mental health services: limited or unavailable services, fear of contracting COVID‐19, perceived high medical costs, transportation challenges, and lack of time. These barriers contributed to delays in the diagnosis and treatment of underlying conditions in Pacific communities (Nosa et al. 2023). At the household level, families adapted to new routines, supported each other emotionally, and found creative ways to stay connected. Young learners struggled with remote learning, especially when they came across challenging concepts or content (Education Review Office 2020). Even when parents took on dual roles as caregivers and educators, many lacked confidence in supporting their children's remote learning (Ministry of Health 2021). For Pacific learners, online learning was less effective than face‐to‐face education (Education Review Office 2020). In the 2021 Delta outbreak, Pacific learners were disproportionately affected and were often from families with COVID‐19 cases, hospitalisations, or deaths. The impact of these losses was intensified by restrictions that prevented community gatherings and traditional funeral practices (Education Review Office 2020).

Strengthening an “all‐of‐community” approach, through partnerships between Pacific organisations, churches, communities, and government agencies, remains essential for improving outreach and service delivery (Ioane et al. 2021; Smith, et al. 2021; Te Poutoko Ora a Kiwa 2024). Treating Pacific providers as equal partners in decision‐making fostered greater community trust and produced more effective responses, while the early involvement of Pacific leadership ensured initiatives were closely aligned with community needs and realities.

### 3.2 Pacific‐Centred Approaches

Pacific‐led responses were characterised by their early mobilisation and reliance on culturally grounded practices, including the establishment of Pacific‐specific emergency teams, church‐based outreach, and the use of talanoa (a traditional Pacific method of open dialogue), to facilitate relational engagement (Ministry for Pacific Peoples 2021; Smith, et al. 2021; Te Hiringa Mahara – Mental Health and Wellbeing Commission 2023). These approaches were vital for building trust, addressing misinformation, and overcoming systemic barriers (Pasifika Proud 2021; Royal Commission 2024). They were often led by trusted figures, including elders, church ministers, Pacific nurses, doctors, and community leaders, who provided practical assistance alongside spiritual and emotional care, reinforcing holistic models of wellbeing (Ministry for Pacific Peoples 2021; Smith, et al. 2021; Te Hiringa Mahara – Mental Health and Wellbeing Commission 2023).

Qualitative accounts from Pacific health professionals and community leaders by Reddy and colleagues (2023) identified four constraints in the national response: the absence of culturally responsive planning within dominant health institutions; inadequate resourcing of Pacific‐led initiatives; the persistence of institutional racism; and the fragmentation of pandemic responses. Participants in the study also described a disconnect between mainstream service provision and the lived realities of Pacific communities, which contributed to low engagement and growing mistrust. Reddy's research argues that effective responses require more than cultural competence within existing structures; it requires a fundamental shift toward community‐led leadership, long‐term investment, and systems intentionally designed to reflect and uphold Pacific knowledge systems and ways of connecting. Rather than serving as supplementary to the mainstream system, Pacific providers and its Pacific workforce were found to be linguistically, culturally, and relationally responsive in ways that mainstream services were not (Reddy et al. 2023; Smith, et al. 2021; Te Hiringa Mahara – Mental Health and Wellbeing Commission 2023).

While national COVID‐19 strategies largely centred on measures such as lockdowns, curfews, hand hygiene, and mask use, Pacific churches demonstrated the added value of embedding spiritual, emotional, and practical support within these measures, reinforcing the importance of holistic, culturally grounded approaches to pandemic preparedness (Ministry for Pacific Peoples 2021; Moana Connect 2024; Te Hiringa Mahara – Mental Health and Wellbeing Commission 2023).

### 3.3 Clear and Trusted Communications

Communication played a vital role in how Pacific communities responded to the pandemic. Pacific organisations and church leaders developed their own communication strategies to ensure health information was both accessible and culturally resonant. For instance, the Ministry for Pacific Peoples released daily bulletins to the Pacific community, which became a key communication focal point (Ratuva et al. 2021). Its website had translated COVID‐19 material in nine languages, including Cook Islands Māori, Vagahau Niue, Tokelauan, Tuvaluan, Samoan, Tongan, Fijian, Rotuman, and Kiribati (Te Hiringa Mahara – Mental Health and Wellbeing Commission 2023). Churches contextualised public health messages through faith‐based framing, such as “stay safe with Jesus in our bubbles,” and the PreparePacific.nz platform, delivered health updates in Pacific languages including Gagana Sāmoa, Lea Faka‐Tonga, Cook Islands Māori, and Vagahau Niue (Ioane et al. 2021; Royal Commission 2024). These messages were not simply translated but were reframed using culturally meaningful language, imagery, and references that aligned with Pacific values of family, faith, and collective wellbeing (Royal Commission 2024). They were disseminated through trusted and culturally relevant channels that existed prior to COVID‐19, such as radio, social media, print media, and online church services, enhancing their reach and credibility among Pacific communities (Ioane et al. 2021; Ministry of Health 2021; Pasifika Proud 2021). A survey of Pacific communities found that the most useful sources of COVID‐19 information were TV news (66%), online platforms (41%), and radio (28%). Distinct age‐related patterns were evident, with television and radio proving more popular among older Pacific people, while younger generations favoured online sources (Brunton 2021b; Ministry of Health 2021).

Church services played a vital role in providing information and emotional support for elders and families in isolation, yet significant barriers remained in ensuring the effective delivery of accurate information (Ministry of Health 2021; Ministry for Pacific Peoples 2021; Pasifika Proud 2021). These included limited access to digital devices and internet connectivity among Pacific families, the loss of valued face‐to‐face engagement, a lack of translated resources, and public health messaging that failed to reflect Pacific values, worldviews, and communication preferences (Brunton 2021a; Ioane et al. 2021; Royal Commission 2024). These challenges added to widespread misinformation and a lack of trust in government messaging. Misinformation, particularly on social media, was a major barrier to vaccine uptake (Brunton 2021a). To counter vaccine hesitancy, reports by Matada Research (2023) and Te Poutoko Ora a Kiwa (2024) noted community‐led methods, such as talanoa, were successful in building trust and addressing misinformation. These conversations, led by Pacific leaders, linked health and scientific information with cultural values such as protecting genealogy and communal wellbeing, making vaccine messaging more relevant and trusted.

To improve future pandemic planning, recommendations from authors have included codesigning messaging with Pacific communities, working alongside Pacific media to deliver information in Pacific languages through trusted channels like churches and youth groups, and investing in digital equity programmes to address access barriers. Strengthening partnerships between government and Pacific providers and building the capacity of Pacific health and social service organisations, are also considered critical to ensuring that communication during future pandemics and emergencies is culturally appropriate, timely, and accessible (Ioane et al. 2021; Moana Connect 2024; Pasifika Proud 2021; Royal Commission 2024; Smith, et al. 2021; Te Poutoko Ora a Kiwa 2024).

### 3.4 Digital Inclusion and Literacy Skills

COVID‐19 accelerated a digital shift that fundamentally reshaped how Pacific communities accessed essential services. Pacific women interviewed in Auckland shared that during and after the COVID‐19 pandemic, learning new technologies to stay connected with family overseas and across Aotearoa was a highly positive and empowering experience (Su’a‐Tavila 2020; Pihigia 2021). Church leaders also observed an increased attendance and stronger family connexions as church activities shifted from the pulpit into family homes and living rooms (Ministry for Pacific Peoples 2021). Young people supported elders and congregations in using technology, enabling access to online church services. These virtual services provided an opportunity to maintain spiritual and emotional well‐being, especially among vulnerable groups such as older adults, widows, and single parents.

While there were positive outcomes for some, digital exclusion remained a significant barrier across all age groups. Many families and the elderly lacked internet access, digital devices, or the skills needed to navigate online platforms. This limited their ability to engage in online learning, access telehealth services, receive health information, and stay informed about Government updates (Ministry of Health 2021; Ministry for Pacific Peoples 2021; Nosa et al. 2023; Pasifika Proud 2021; Ratuva et al. 2021). Pacific organisations and churches responded by initiating digital skills programmes such as DIGIFALE, where Pacific youth partnered with elders to build confidence using mobile phones. These sessions focused on practical skills, such as booking and managing health appointments, and accessing essential services online (Matenga‐Ikihele et al. 2023).

Digital equity was identified as an important structural determinant of public health (Moana Connect 2024). Key recommendations include the codesign of digital solutions in genuine partnership with Pacific communities, the delivery of digital literacy initiatives through trusted community channels (e.g. churches and youth groups), and the provision of equitable access to digital infrastructure, including devices and internet connectivity. Authors have suggested that long‐term investment in digital infrastructure and capacity‐building for Pacific health and social service providers will be critical in strengthening future pandemic preparedness. Enhancing digital health literacy, particularly within Pacific communities, will be critical in preventing the spread of misinformation and enable individuals and families to effectively assess health information and make informed decisions, including around vaccine uptake (Royal Commission 2024; Moana Connect 2024).

### 3.5 Economic Support and Sustainability

Economic support during the COVID‐19 pandemic included both government‐led and community‐driven initiatives, such as the COVID‐19 wage subsidy, WINZ hardship grants, and IRD‐administered business and income support schemes (Nosa et al. 2023). One of the most notable enablers of economic support for Pacific communities was the Government's targeted COVID‐19 recovery package, which allocated NZ$195 million specifically for Pacific communities. This funding extended beyond immediate relief to include investments in education, housing, employment, and cultural heritage (Ratuva et al. 2021; Sio 2020). Alongside broader wage subsidy schemes, these measures were implemented to buffer against widespread job losses and business closures. When resources were filtered through procurement channels, many community organisations and leaders were sidelined, despite being best placed to understand and respond to local needs. As a result, some frontline providers struggled to access adequate funding, creating bottlenecks in service delivery. Churches, Pacific health and social service providers, and community organisations frequently stepped in to ‘gap‐fill’, with unpaid labour, short‐term grants, or personal resources to meet urgent needs such as food distribution, wellbeing checks, and translation of health information (Pasefika Proud 2021). This exposed systemic underinvestment in Pacific‐led infrastructure and highlighted the innovation of Pacific providers, churches and community organisations.

Pacific families experienced increased economic pressures during lockdowns, driven by increased household food costs when school lunches were no longer available, and additional family members joined household bubbles (Education Review Office, 2020; Ministry of Health 2021; Ratuva et al. 2021). A survey of Pacific respondents living in South Auckland found almost one in five (18%) Pacific households lost half or more of their household income due to COVID‐19 (Brunton 2021b). These findings were consistent in a separate survey that found many experienced a decline in household income (80%), job losses (25%), and some form of psychological distress (over 50%). Despite the significant increase in the number of Pacific communities who accessed Jobseeker benefits during COVID‐19 (Ratuva et al. 2021), a large proportion felt embarrassed to seek financial assistance (70%), were unaware of available support (65%), or were hesitant to apply due to uncertainty about their rights (64%) (Nosa et al. 2023).

Some households adopted sustainable living practices, such as community gardening and shared cooking, to support wellbeing and food security (Matada Research 2023; Royal Commission 2024). Churches, while instrumental in providing outreach and care, were also identified as sites of tension. One report noted that some families felt expected to continue making financial contributions (e.g. tithes) during a period of significant financial strain. This finding highlights the complex interplay between cultural and faith obligations, particularly within contexts of economic precarity (Ratuva et al. 2021). Many senior Pacific learners took on family responsibilities such as caring for siblings or entered part‐time or full‐time employment, often in essential service roles, to provide financial support for their families. While this demonstrated a strong sense of familial responsibility, the dual burden of work and schooling presented substantial challenges, with some students deprioritising their learning or leaving school altogether (Education Review Office 2022). It also highlighted the overrepresentation of Pacific communities in lower‐income sectors, particularly service industries such as healthcare, social assistance and labouring roles in manufacturing, transport, warehousing, and logistics (Ministry of Health 2021; Nosa et al. 2023).

Findings from this review indicate a need for strengthened strategic and financial commitments to support Pacific‐led emergency responses. Key recommendations highlighted across the documents included streamlining funding pathways, improving access to essential supplies, and building community economic resilience through locally tailored initiatives. Underpinning these recommendations is the need for long‐term funding models that direct decision‐making abilities to Pacific providers (Moana Connect 2024; Te Poutoko Ora a Kiwa 2024). Embedding economic support within culturally responsive frameworks, strengthening government‐community partnerships, and addressing structural inequities were identified as critical steps toward equitable and effective pandemic preparedness and recovery.

## 4 Pandemic Preparedness Aspirations

Lessons from COVID‐19 highlight aspirations and recommendations to strengthen the ability of Pacific communities, providers, and churches to respond effectively in future crises. Achieving this requires a whole‐of‐society approach to pandemic preparedness, recognising that success depends on the combined efforts of all sectors working alongside individuals, families, and communities. A proposed approach combines the culturally grounded governance of the Soalaupule Ecosystem Framework (Ministry of Health 2023) with the staged, values‐based operations of the Wansolwara Pacific Public Health Emergency Response Framework (Moana Connect 2024), offering a model that is community‐led in decision‐making and culturally grounded.

**FIGURE 2:**
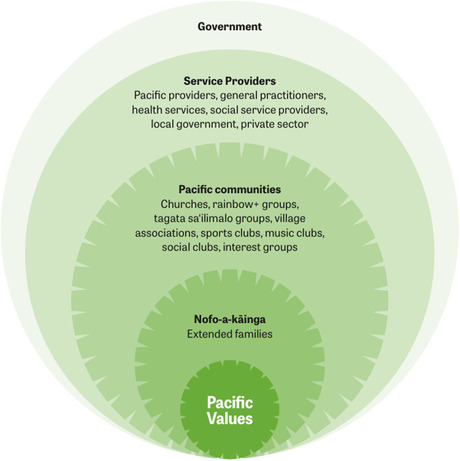
The Soalaupule Ecosystem Framework, developed by Tunumafono Fa‘amoetauloa Avaula Fa‘amoe (Ministry of Health 2023) reflects the collective nature of Pacific communities and support systems, highlighting the inclusive approach required for effective pandemic preparedness planning for Pacific communities.

The Soalaupule Ecosystem Framework (Figure 2), developed by Tunumafono Fa‘amoetauloa Avaula Fa‘amoe, reflects the collective nature of Pacific communities, where support systems are grounded in relationships and decisions are made through inclusive, consensus‐driven processes. Rooted in values of family, respect, spirituality, and love, it uses talanoa to engage diverse voices, including marginalised groups, building trust, aligning priorities, and formalising the role of natural support systems such as extended families, churches, village associations, and sports clubs in shaping and delivering preparedness measures (Ministry of Health 2023).

The Wansolwara Framework translates these shared priorities into a clear, three‐stage operational pathway specific to delivering a public health emergency response: Mateuteu (preparedness), Anga Lelei (response), and Ako (learning). For the preparedness stage, Wansolwara provides specific, role‐based actions for families, churches and community groups, Pacific health and social service providers, and regional/national agencies. These include developing detailed emergency plans, building surge capacity, securing essential supplies, establishing communication systems, and formalising agreements with partner agencies (Moana Connect 2024).

By merging these frameworks, preparedness moves beyond compliance with generic emergency management templates to become a living, community‐owned process. Soalaupule principles guide how decisions are made, through inclusive, respectful, and consensus‐based governance; while Wansolwara defines what needs to be done, by whom, and with what resources. This integration ensures that (1) plans are codesigned and trusted, enabling rapid activation when crises arise; (2) roles and responsibilities are clear, reducing duplication and delays in mobilisation; (3) communication channels are pre‐established, ensuring culturally tailored health messages reach every household; (4) resources are matched to community priorities, with flexible funding and scalable infrastructure ready for deployment; and (5) preparedness is continuously strengthened through annual simulations, skills training, and the Ako stage's structured reflection.

Embedding this merged approach within Pacific communities’ preparedness aspirations will create a system that is locally led, nationally supported, and culturally anchored, capable of responding effectively not only to future pandemics but to any public health emergency. These preparedness aspirations are presented in Table 2, bringing together the inclusive governance principles of the Soalaupule Ecosystem Framework and the staged actions of the Wansolwara Pacific Public Health Emergency Response Framework. Each aspiration outlines specific actions, identifies responsible actors across community, service, and system levels, and aligns with the relevant Wansolwara stages (Mateuteu, Anga Lelei, Ako) to deliver a culturally grounded, coordinated, and continually improving response.

**TABLE 2:** Pacific pandemic/emergency preparedness aspirations and implementation responsibilities.

Preparedness Aspiration	Details	Responsibility	Area	Wansolwara Stages
**Strengthen Pacific community partnerships**	Develop church‐ and community‐led emergency response plans that include food security, financial assistance, and spiritual support. Ensure continuity of learning and cultural connexion for Pacific learners and equitable digital access. Strengthen interagency collaboration between Pacific stakeholders and government agencies to streamline service delivery and funding. Ensure Pacific providers, workforce, churches and community leaders are engaged as equal partners at the outset of planning and implementation. Adapt commissioning processes to support Pacific ways of working and equitable resource allocation.	Government (MoH, MoE); Service Providers (Pacific health providers, regional authorities); Pacific communities (churches, community groups, community leaders).	Local/ National/ Regional	Mateuteu/ Anga Lelei/ Ako
**Embed Pacific centred approaches**	Ensure all protective measures (e.g., visitation policies) are culturally safe. Frame vaccination and health messaging around family, church, and social wellbeing. Support Pacific‐led education plans that reflect intergenerational and collective living realities. Leverage churches to support recovery efforts.	Government (MoH, MoE); Service Providers (Pacific health providers, regional authorities); Pacific communities (churches, community groups, community leaders).	National/ Local	Mateuteu/ Anga Lelei
**Deliver clear and trusted communication**	Develop communication strategies that reflect Pacific languages, ages, values, and channels (social media or radio) to improve message uptake and counter misinformation. Use trusted community messengers (e.g., church leaders, elders, youth) to share timely and culturally tailored public health messages.	Government (MoH); Service Providers (Pacific health providers, regional authorities, local government); Pacific communities (churches, community groups, Pacific media).	National/ Regional	Mateuteu/ Anga Lelei/ Ako
**Advance digital inclusion and health literacy**	Address digital inequities early, especially for intergenerational households. Invest in devices, internet access, and digital skills training. Support digital health literacy to enable Pacific families to confidently access telehealth, health updates, and online services.	Government (MoE, MBIE, MoH); Service Providers (Pacific education providers, regional authorities, local government, TELCOs); Pacific communities (churches, community groups).	National/ Local	Mateuteu/ Ako
**Secure economic support and sustainability**	Establish accessible funding pathways for Pacific‐led health, church, social services and community providers to activate during emergencies. Recognise the burden on Pacific essential workers and provide tailored health and wellbeing support. Ensure sustained funding for Pacific‐led health, church, and social services. Improve systems for equitable and needs‐based distribution of resources.	Government (MSD, MoH, Treasury); Service Providers (Pacific health providers, regional authorities, local government); Pacific communities (churches, community groups).	National/ Regional	Mateuteu/ Anga Lelei/ Ako

## 5 Discussion

This review examined the response of Pacific communities in Aotearoa New Zealand to the COVID‐19 pandemic, integrating findings from academic literature, government reports and community‐led research published between 2020 and 2024. Five interconnected themes were identified: community partnerships; Pacific‐centred approaches; clear and trusted communication; digital inclusion; and economic support. Each theme reveals critical enablers, barriers, and opportunities for improved future pandemic preparedness.

The most impactful enablers were long‐standing partnerships between Pacific providers, churches, communities, and, in some cases, government agencies; the use of culturally grounded communication channels and trusted messengers; agile coordination within church and community structures; and intergenerational collaboration that bridged digital divides. These mirror Wansolwara's Mateuteu stage priorities of relationship‐building, clear communication pathways, and preparedness planning that is both community‐led and culturally anchored.

While positive, these achievements were sustained largely through unpaid labour, short‐term grants, and overstretched volunteer networks, exposing structural vulnerabilities and risking burnout (Moana Connect 2024; Royal Commission 2024). Under‐resourced organisations were unable to maintain operations over prolonged periods, and Pacific leaders were not consistently included in decision‐making from the outset, limiting alignment with community realities (Ioane et al. 2021; Te Poutoko Ora a Kiwa 2024). Persistent digital inequities, inadequate translation services, and structural economic disadvantages compounded these barriers, disproportionately affecting Pacific people in low‐wage, high‐risk employment sectors (Brunton 2021b; Nosa et al. 2023; Ratuva et al. 2021; Ministry for Pacific Peoples 2021).

These findings reinforce that resilience without investment is not sustainable. Future preparedness must formally integrate Pacific‐led responses into emergency management systems, supported by long‐term, high‐trust, flexible funding, and co‐governance mechanisms (Ratuva et al. 2021; Te Hiringa Mahara – Mental Health and Wellbeing Commission 2023). Consistent with the Soalaupule Ecosystem Framework (Ministry of Health 2023), national and regional agencies should embed Pacific leadership from the planning stage through to implementation, ensuring equitable access to resources and authority in decision‐making.

A notable gap in the literature was the absence of children's and young people's perspectives. While several articles acknowledged the role of Pacific youth, often through learning experiences, assisting churches with food distribution, or supporting elders with technology, there was little examination of their specific needs, priorities, or viewpoints. Insights into Pacific young people's experiences were largely limited to broader research, such as the Growing Up in New Zealand COVID‐19 Wellbeing Survey (Meissel et al. 2021). Although research shows children were less likely than older adults to contract COVID‐19 or experience severe symptoms (Irwin, et al. 2022), Pacific children and youth still experienced significant disruptions to education, reduced social interaction, increased caregiving responsibilities, and psychological impacts. The Australian and New Zealand Paediatric Infectious Diseases (ANZPID) Group of the Australasian Society for Infectious Diseases (ASID) calls for the urgent need to include children's needs and voices in future pandemic planning, a priority that should also be recognised for Pacific communities in Aotearoa New Zealand (Campbell et al. 2022).

This article confirms that Pacific pandemic resilience is built on culturally anchored leadership, relational trust, and agile, community‐driven systems. To sustain and strengthen these capacities, this review recommends consistent structural investment, formal inclusion in governance, and long‐term capacity building. Pacific conceptions of wellbeing are inherently holistic, encompassing physical health, relationships, spirituality, and community (Pulotu‐Endemann 2001). This understanding highlights that preparedness frameworks move beyond biomedical models to embed Pacific values and collective approaches at every stage, from governance to service delivery and workforce. The Soalaupule Ecosystem Framework and the Wansolwara Framework together offer a culturally aligned blueprint for institutionalising collaborative leadership, shared accountability, and relational decision‐making in pandemic preparedness. Integrating Pacific‐led, culturally grounded systems into national planning will strengthen equity, foster trust, and enhance the overall resilience of Aotearoa's public health response. Without such systemic change, future crises risk repeating the under‐resourced and inequitable responses experienced during COVID‐19.

## 6 Conclusion

The experiences of Pacific communities during the COVID‐19 pandemic demonstrate that an effective response was driven by culturally anchored leadership, relational trust, and the mobilisation of agile, community‐based systems. These strengths enabled rapid, coordinated action that met immediate needs while sustaining social cohesion and well‐being. However, the pandemic also exacerbated existing inequities and exposed additional systemic vulnerabilities, including underinvestment, reliance on unpaid labour, fragmented decision‐making, and the persistent exclusion of Pacific leadership from formal governance structures.

Moving forward, preparedness must be reframed to position Pacific‐led systems as a core part of Aotearoa's public health infrastructure. The Soalaupule Ecosystem Framework and the Wansolwara Framework together offer a culturally grounded pathway for embedding collaborative leadership, shared accountability, and relational decision‐making across all stages of emergency management. By committing to long‐term capacity building, ensuring equitable resource allocation, strengthening digital inclusion, and institutionalising Pacific co‐governance, Aotearoa New Zealand can create a pandemic response system that is more equitable, trusted, and resilient. Central to this is the active inclusion of children and young people within these frameworks, ensuring their perspectives and needs help shape decisions. Such engagement will strengthen equity, deepen trust, and enhance resilience. Without these structural changes, future crises risk deepening the inequities and repeating the under‐resourced responses witnessed during COVID‐19.

Strengths of this review include its systematic approach to searching and collating evidence on Pacific responses to COVID‐19 in Aotearoa, New Zealand. While the review draws primarily on published research and formal reports, this reliance may have limited the visibility of locally grounded, informal, or community‐led responses that are less frequently published.

## Funding

This study was supported by Te Niwha, the Infectious Diseases Research Platform – co‐hosted, and Ministry of Business, Innovation and Employment, New Zealand. The funders had no role in the design and conduct of the study; data collection, management, analysis, and interpretation; manuscript preparation or review; or the decision to submit the manuscript for publication.

## Conflicts of Interest

The authors declares no conflicts of interest.

## Data Availability

The data that support the findings of this study are available from the corresponding author upon reasonable request.

## References

[snz270049-bib-0002] Brunton, C. 2021a. Attitudes towards COVID‐19 Vaccination Amongst Pacific Peoples Ministry of Health. https://www.health.govt.nz/system/files/2021‐09/pacific_peoples_covid‐19_vaccination_research_report_proactive_release.pdf.

[snz270049-bib-0003] Brunton, C. 2021b. Impact of COVID‐19 on Pacific People Living in South Auckland. Ministry of Health. https://www.health.govt.nz/system/files/2021‐03/impact_of_covid‐19_on_pacific_peoples_living_in_south_auckland.pdf.

[snz270049-bib-0001] Campbell, A. J. , A. M. Ranzoni , E. A. Clutterbuck , R. Booy , and H. Nohynek . 2022. “Widening the Lens for Pandemic Preparedness: Children Must Be Seen and Heard.” The Lancet Regional Health – Western Pacific 51: 101205. 10.1016/j.lanwpc.2021.101205. PMC11424934 39328247

[snz270049-bib-0004] Education Review Office . 2022. Learning in a Covid‐19 World: The Impact of Covid‐19 on Pacific Learners. Education Review Office. https://evidence.ero.govt.nz/media/53tjuapp/impact‐of‐covid‐on‐pacific‐learners‐report.pdf.

[snz270049-bib-0005] Ioane, J. , T. Percival , W. Laban , and I. Lambie . 2021. “ALl‐of‐Community by ALl‐of‐Government: Reaching Pacific People in Aotearoa New Zealand during the COVID‐19 Pandemic.” The New Zealand Medical Journal 134, no. 1533: 96–103. 33927427

[snz270049-bib-0006] Irwin, M. , B. Lazarevic , D. Soled , and A. Adesman . 2022. “The COVID‐19 Pandemic and Its Potential Enduring Impact on Children.” Current Opinion in Pediatrics 34, no. 1: 107–115. 10.1097/MOP.0000000000001097. 34923563 PMC8728751

[snz270049-bib-0007] Matada Research . 2023. The $7 Cabbage Dilemma: Pacific Food Security and Resilience during COVID‐19. Matada Research. https://matadaresearch.co.nz/wp‐content/uploads/The‐7‐cabbage‐dilemma‐Pacific‐peoples‐New‐Zealands‐COVID‐19‐response.pdf.

[snz270049-bib-0008] Matenga‐Ikihele, A. , F. Fa’alau , R. Dobson , et al. 2023. “Navigating Digital Inclusion and the Digital vā among Niue Mamatua through the Provision of Mobile Phones during COVID‐19.” AlterNative: An International Journal of Indigenous Peoples 19, no. 1: 145–154. 10.1177/11771801221148343 PMC9902779 38603307

[snz270049-bib-0030] Meissel, K. , M. Bergquist , J. Kumarich , et al. 2021. “The Growing Up in New Zealand COVID-19 Wellbeing Survey: Part 2: Education. Auckland: Growing Up in New Zealand.”

[snz270049-bib-0009] Ministry for Pacific Peoples . 2021. Pacific Churches and COVID‐19: Community Response Report. Wellington. https://www.mpp.govt.nz/assets/Reports/MPP_PacificPeoplesCOVID2020web.pdf.

[snz270049-bib-0010] Ministry for Pacific Peoples . 2022. Yavu – Foundations of Pacific Engagement Ministry for Pacific Peoples. https://www.mpp.govt.nz/assets/Resources/Yavu‐Booklet.pdf.

[snz270049-bib-0011] Ministry of Health . 2021. Pacific Families and Frontline Workers’ Experience of COVID‐19. Wellington. https://www.health.govt.nz/system/files/2021‐06/research_report_qualitative_study_28may_redacted_watermarked.pdf.

[snz270049-bib-0012] Ministry of Health . 2023. Te Mana Ola: The Pacific Health Strategy 2023. Wellington.

[snz270049-bib-0013] Ministry of Health . 2025. Tupa Ola Moui: Pacific Health Chart Book 2025: Volume 1: Pacific Population in New Zealand Ministry of Health. www.health.govt.nz/system/files/2025‐05/tupu‐ola‐moui‐volume‐1‐pacific‐population‐new‐zealand‐v2.pdf.

[snz270049-bib-0014] Moana Connect . 2024. Wansolwara Framework: Pacific‐Led Emergency Response and Wellbeing. Auckland.

[snz270049-bib-0015] Nosa, V. , J. Sluyter , A. Kiadarbandsari , et al. 2023. “Service Uptake Challenges Experienced by Pasifika Communities during COVID‐19 Lockdowns in New Zealand.” COVID 3, no. 11: 1688–1697. 10.3390/covid3110116.

[snz270049-bib-0016] Pasifika Proud . 2021. Prepare Pacific: Insights Report on Pacific COVID‐19 Responses Ministry of Social Development.

[snz270049-bib-0017] Pihigia, M. M. 2021. Hidden Talents: Taleni Tanumia Mafola Press.

[snz270049-bib-0018] Pulotu‐Endemann, F. K. 2001. Fonofale Model of Health https://bit.ly/3tIlQNA.

[snz270049-bib-0019] Ratuva, S. , Y. Crichton‐Hill , T. Ross , A. Basu , P. Vakaoti , and R. Martin‐Neuninger . 2021. “Integrated Social Protection and COVID‐19: Rethinking Pacific Community Responses in Aotearoa.” Journal of the Royal Society of New Zealand 51, S37. 10.1080/03036758.2020.1861033.

[snz270049-bib-0020] Reddy, R. , J. Sluyter , A. Kiadarbandsari , et al. 2023. “Priority Health Needs and Challenges in New Zealand Pacific Communities‐A Qualitative Analysis of Healthcare Delivery during the COVID‐19 Pandemic.” Healthcare (Basel, Switzerland) 11: 2239. 10.3390/healthcare11162239. 37628437 PMC10454131

[snz270049-bib-0021] Royal Commission . 2024. Royal Commission of Inquiry into COVID‐19 Lessons Learned: Pacific Communities Report. Royal Commission. whttps://www.covid19lessons.royalcommission.nz/reports‐lessons‐learned/main‐report/.

[snz270049-bib-0022] Sio, A. 2020. Supporting Pacific Peoples through the COVID‐19 Recovery Plan [Press Release] New Zealand Government. https://www.beehive.govt.nz/release/supporting‐pacific‐peoples‐through‐covid‐19‐recovery‐plan.

[snz270049-bib-0023] Steyn, N. , R. Binny , K. Hannah , et al. 2020. “Māori and Pacific People in New Zealand Have Higher Risk of Hospitalisation for COVID‐19.” medRxiv 10.1101/2020.12.25.20248427. 34239143

[snz270049-bib-0024] Smith, A. , S. Fereti , and S. Adams . 2021. “Inequities and Perspectives from the COVID‐Delta Outbreak: The Imperative for Strengthening the Pacific Nursing Workforce in Aotearoa New Zealand.“ Nursing Praxis in Aotearoa New Zealand 37, no. 3: 94–103. 10.36951/27034542.2021.040.

[snz270049-bib-0025] Su’a‐Tavila, A. , T. B. Pereira , and M. T. Manuleleua . “The Experience of Pacific Women in Auckland during and Post the COVID‐19 Pandemic.” Accessed 2020. https://www.women.govt.nz/sites/default/files/2022‐04/4578_MFW_Pacific%20Women%20Covid%20Report_v3.2%20KW.pdf.

[snz270049-bib-0026] Te Hiringa Mahara – Mental Health and Wellbeing Commission . 2023. Pacific Connectedness and Wellbeing in the Pandemic. Te Hiringa Mahara - Mental Health and Wellbeing Commission. https://www.mhwc.govt.nz/assets/Reports/COVID‐19‐series/Paper‐7/COVID‐19‐paper‐7‐Full‐report.pdf.

[snz270049-bib-0027] Te Poutoko Ora a Kiwa . 2024. Final Report Summary: COVID‐19 and National Immunisation Programme Research. https://bpb‐ap‐se2.wpmucdn.com/blogs.auckland.ac.nz/dist/4/894/files/2025/10/prop‐007‐final‐summary‐report.pdf.

[snz270049-bib-0029] Te Whatu Ora . 2022. Ola Manuia: Interim Pacific Health Plan July 2022–June 2024. Te Whatu Ora – Health New Zealand. Available from https://www.tewhatuora.govt.nz/assets/Publications/Ola‐Manuia‐iPHP‐A4.pdf.

[snz270049-bib-0028] World Health Organization . 2020. WHO Interim Guidance: Risk Communication and Community Engagement for Novel Coronaviruses (nCoV World Health Organization. Switzerland. https://coilink.org/20.500.12592/f4spdx.

